# Renal Infarction Following Anticoagulant Discontinuation in a Pediatric Patient With Congenital Heart Disease

**DOI:** 10.7759/cureus.80540

**Published:** 2025-03-13

**Authors:** Yusuke Asami, Yuko Kajiho, Akiko Kinumaki, Kazuhiro Shiraga, Ryo Inuzuka

**Affiliations:** 1 Department of Pediatrics, The University of Tokyo, Tokyo, JPN

**Keywords:** anticoagulants, cardiogenic risk, congenital heart disease, fontan surgery, pediatric patients, renal infarction

## Abstract

Renal infarctions are rare in pediatric patients. While cardiogenic risk has been documented in adults, the association between renal infarction and congenital heart disease in children is seldom reported. Consequently, the pathogenesis and risk remain unclear. This report aims to highlight a potential association between anticoagulant discontinuation and renal infarction in pediatric patients with congenital heart disease. Herein, we present the case of a nine-year-old boy who underwent Fontan surgery for a left-sided, morphologic right ventricle and for whom anticoagulants were discontinued following oral bleeding. The patient presented with sudden abdominal pain and was diagnosed with renal infarction based on his medical history and contrast-enhanced CT findings. Serum D-dimer levels were later found to be elevated. Anticoagulant discontinuation in patients with congenital heart disease is a risk factor for renal infarction, necessitating intensified monitoring. In at-risk patients, renal infarction should be considered in the differential diagnosis of acute abdomen.

## Introduction

Renal infarction is a rare condition, accounting for 0.004%-0.007% of emergency visits [1,2]. In a study involving 438 adult patients with renal infarction, causes were cardiogenic (n=244 (55.7%)), renal artery injury (n=33 (7.5%)), coagulopathy (n=29 (6.6%)), and idiopathic (n=132 (30.1%)) [3]. Among the cardiogenic causes, atrial fibrillation accounted for approximately half of these cases, followed by cardiomyopathy and prosthetic valves [3].

Reports on renal infarction in pediatric patients are even more limited. A search of the PubMed database revealed only 10 pediatric non-traumatic renal infarction cases associated with renal vascular problems or systemic diseases, including multisystem inflammatory syndrome in children [4-12]. Furthermore, reports of cardiogenic causes of renal infarction in children are rarer than those in adults, and very few cases have been reported to be associated with congenital heart disease [12]; therefore, the risk factors and pathogenesis of this disease remain unclear.

This report presents a case of a pediatric patient who underwent a Fontan procedure for congenital heart disease. The Fontan circulation is associated with a high risk of thrombosis, necessitating long-term antithrombotic therapy. However, due to the rarity of patients with a single-ventricle and thrombotic events, evidence-based guidelines remain limited, preventing large-scale definitive clinical trials.

In this report, we describe a pediatric case of renal infarction following anticoagulant discontinuation after the Fontan procedure for congenital heart disease, highlighting the potential risks associated with the discontinuation of anticoagulants in such patients.

## Case presentation

A nine-year-old boy presented to our ED with fever, abdominal pain, and vomiting. The patient underwent Fontan surgery for a left-sided, morphologic right ventricle, and an extended polytetrafluoroethylene conduit (intra-extracardiac conduit) was used to connect the hepatic vein to the pulmonary artery via the right atrium. The patient had pulmonary atresia with a significantly hypoplastic main pulmonary artery, which was not blind-ended. The patient also underwent pacemaker implantation for congenital atrioventricular heart block. No shunts, such as veno-venous shunts or pulmonary arteriovenous fistulas, had been identified, and the patient did not have a fenestration. There was no history of atrial fibrillation. Given the high risk of thrombosis associated with the conduit, warfarin had been used as antithrombotic therapy until this hospital visit. Six weeks prior to admission, warfarin was discontinued because of mucosal bleeding following an oral procedure.

The day before admission, the patient experienced sudden abdominal pain and vomiting. The next day, he developed fever and right-sided back pain and visited his previous physician. Contrast-enhanced CT revealed extensive non-contrast areas consistent with the dorsal vascular region of the right kidney, and hence, he was referred to our hospital. He had no family history of urinary tract infections or congenital anomalies of the kidneys or urinary tract. The patient was administered enalapril maleate 6 mg/day for his heart.

The patient was conscious and lucid, with a temperature of 37.7°C, heart rate of 72/min, no atrial fibrillation, blood pressure of 95/58 mmHg, and SpO2 of 99% in room air. Physical examination revealed no abnormal findings in the head, neck, or chest regions. The abdomen was flat and soft with tenderness extending from the pericardial fossa to the umbilical region and pain at the right costovertebral angle. No edema or skin rash was observed on the extremities. There were no signs of dehydration. Blood tests (Table 1) revealed elevated leukocytes with significant neutrophilia, a C-reactive protein level of 4.5 mg/dL, and a lactate dehydrogenase (LDH) level of 765 U/L. As for coagulation, the prothrombin time-international normalized ratio (PT-INR) was 1.62, and D-dimer was in the normal range at the beginning but later increased to 1.4 μg/mL on day 5 and 0.9 μg/mL on day 12 of admission (Table 2).

**Table 1:** Blood test results at the time of admission. ALT: Alanine Aminotransferase; APTT: Activated Partial Thromboplastin Time; AST: Aspartate Aminotransferase; BUN: Blood Urea Nitrogen; CBC: Complete Blood Count; CRP: C-reactive Protein; eGFR: Estimated Glomerular Filtration Rate; Hb: Hemoglobin; Hct: Hematocrit; LDH: Lactate Dehydrogenase; PT: Prothrombin Time; INR: International Normalized Ratio.

Parameter	Result
WBC	21,200/μL
Neutrophils	90.90%
RBC	5.27 × 10^4^ /μL
Hb	14.6 g/dL
Hct	43.80%
Platelet	218 × 10^4^ /μL
Albumin	3.9 g/dL
AST	93 U/L
ALT	94 U/L
LDH	765 U/L
BUN	9.2 mg/dL
Creatinine	0.31 mg/dL
eGFR	147 mL/min/1.73 m^2^
Sodium	130 mEq/L
Potassium	4.0 mEq/L
Chloride	96 mEq/L
CRP	4.45 mg/dL
PT	41.60%
PT-INR	1.62
APTT	34.5 sec
D-dimer	<0.5 μg/mL

**Table 2:** Changes in D-dimer levels after hospitalization. D-dimer levels were initially negative but later increased and subsequently decreased over time.

Day	1	5	12	19
D-dimer (μg/mL)	<0.5	1.4	0.9	0.5

**Figure 1:**
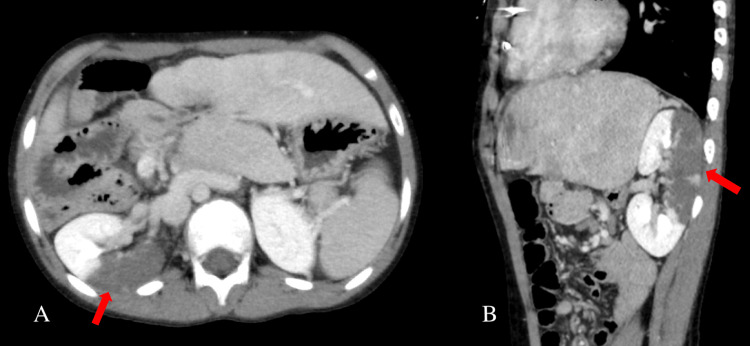
Contrast-enhanced CT of the abdomen at onset shows axial (A) and sagittal (B) views. These images reveal an extensive hypoabsorptive zone on the dorsal surface of the right kidney, with no cortical rim sign. The size of the infarct is 8 cm × 4 cm.

Urine dipstick analysis showed microscopic hematuria but no leukocytes. Renal ultrasonography revealed a hypoechoic area in the dorsal right kidney and decreased Doppler signals. Blood and urine cultures were negative, while echocardiography revealed no intracardiac vegetation. Contrast-enhanced CT revealed an extensive shadow defect in the dorsal right kidney with no rim signs (Figure 1). The size of the infarct was 8 cm × 4 cm.

Contrast-enhanced CT is preferred for diagnosing acute abdomen conditions, such as urolithiasis. Given the patient’s severe pain and inability to tolerate prolonged immobility for MRI, this imaging modality was selected. Differential diagnoses for abdominal pain caused by thromboembolism include mesenteric ischemia, splenic infarction, and hepatic infarction. However, in this case, contrast-enhanced CT aided in ruling out these conditions.

Renal infarction was the primary suspicion; however, based on the time course, endovascular treatment was not indicated, and heparin was initiated as anticoagulation therapy. Initially, acute focal bacterial nephritis could not be ruled out owing to the absence of elevated D-dimer levels; therefore, antibiotic therapy was administered. After admission, the patient developed a persistent high fever, which resolved on the fifth day. The patient's back pain subsided after three days and showed further improvement by the sixth day. Anticoagulation therapy was switched from heparin to oral warfarin due to its ability to be monitored using PT-INR as a treatment indicator. The patient was discharged on day 16 of hospitalization. Since the patient was deemed at high thrombotic risk, anticoagulation therapy was continued after discharge, with a target PT-INR of 2.0-2.5, and there was no recurrence of renal infarction. A follow-up USG performed two months later revealed persistent areas of reduced perfusion, with slight marginal irregularities suggesting the onset of atrophy. The remaining renal tissue had compensated, and renal function remained stable to date. However, a CT scan performed two years after the onset of the disease showed atrophy in the dorsal portion of the right kidney, consistent with post-infarction changes. Long-term deterioration of renal function remains a concern and necessitates careful monitoring.

## Discussion

In the present case, the patient did not present with the cortical rim sign on contrast-enhanced CT, which is specific for renal infarction [13]. The cortical rim sign typically appears approximately 8 hours after onset and has been reported in about 50% of cases; however, some studies suggest that it eventually develops in all cases. While it is considered specific to renal infarction, its actual specificity remains uncertain. In this case, the CT may have been performed before the cortical rim sign became detectable. Given the patient’s pediatric status, repeat imaging during the acute phase was not conducted due to concerns regarding radiation exposure. Additionally, the initial D-dimer level was negative, complicating the diagnosis of renal infarction. In contrast, the sudden onset of disease, lack of evidence of abnormality or increased density of the perirenal fat tissue on contrast-enhanced CT, and the negative results of various culture tests all supported the diagnosis of renal infarction rather than other bacterial infectious diseases [14, 15].

Although there have been few reports summarizing the relationship between renal infarction and D-dimer levels in the PubMed database, even in a prior report indicating the usefulness of D-dimer in renal infarction, the D-dimer was only mildly elevated at 1.1 μg/mL [16]. In pulmonary thromboembolism (PE), it has been reported that PE cannot be ruled out even if D-dimer is negative in cases with a high pretest probability [17], while D-dimer levels are not always initially positive in patients with thromboembolism. Indeed, there are some cases in which D-dimer levels are initially negative. Therefore, renal infarction cannot be ruled out, even if the D-dimer level is normal or low at the onset.

Among cardiogenic infarcts, cases of anomalous embolism associated with a patent foramen ovale or atrial septal defect have been reported in adults; however, very few cases have been reported in children [12]. There have been several prior reports confirming congenital heart disease after treatment, including one of a young adult male patient who neglected to take warfarin following Fontan surgery [18], and one man who increased the dose of diuretics at his own discretion after a Mustard congenital heart surgical procedure for complex cardiac malformations [19]. Both patients required anticoagulants for postoperative thrombophilic treatment of congenital heart disease. Similarly, in the present case, discontinuation of anticoagulants before disease onset was considered to have caused renal infarction. Abnormal coagulation is a known cause of renal infarction [3], and discontinuation of anticoagulants in patients with congenital heart disease, which is considered to have a high tendency for thrombosis due to hemodynamic instability, may increase the risk for renal infarction. In this case, the mechanism of thrombus formation remains unclear, as no apparent shunts or atrial fibrillation was identified. However, given the high thrombotic tendency of the Fontan circulation due to various factors associated with Virchow's triad, it is speculated that the discontinuation of anticoagulants led to coagulation abnormalities, ultimately resulting in thrombus formation.

Traditionally, aspirin and warfarin have been the standard choices for anticoagulation. In recent years, direct oral anticoagulants (DOACs) have also been introduced, demonstrating efficacy [20]; however, further studies and case accumulation are needed to establish their optimal use in this population. It has been reported that the risk of thrombosis after the Fontan procedure is highest from immediately after surgery until six months post-surgery, after which the risk is low but increases and reaches a second peak 10 years after surgery [20]. It has also been reported that the incidence of thromboembolism is 82% (95% CI: 74-87%) 25 years after surgery [20]. In this case, thromboembolism occurred 5 years after surgery. The risk increases as pediatric patients enter adulthood, and more careful monitoring will be necessary.

Particularly in patients with congenital heart disease at high risk of thrombosis-such as those who have undergone Fontan surgery-or in situations that increase thrombosis risk, like discontinuation of anticoagulants, renal infarction should be considered in the differential diagnosis of an acute abdomen, even if D-dimer assessment is negative. In cases where anticoagulation must be discontinued due to an interventional procedure or bleeding, individualized thrombotic risk should be carefully assessed, and PT-INR should be monitored frequently. Once the patient's condition stabilizes, anticoagulation should be promptly resumed to mitigate the risk of thromboembolic events. Additionally, patients should be preemptively informed about their thrombotic risk and potential symptoms to facilitate timely medical evaluation, including contrast-enhanced CT, if symptoms arise. In cases where abdominal pain persists or organ damage is suspected, repeat imaging should be conducted to assess disease progression.

This case report is based on findings from a single case with a two-year follow-up period. Future studies involving further cases are anticipated to investigate the incidence of renal infarction following the Fontan procedure and evaluate its relationship with anticoagulant therapy.

## Conclusions

Interruption of anticoagulants in patients with congenital heart disease, particularly those with a high thrombotic tendency, such as after Fontan surgery, is a risk factor for renal infarction in children. However, few reports on cardiogenic causes in children have been published to date. In at-risk patients, renal infarction should be considered in the differential diagnosis of sudden abdominal pain, even if the D-dimer test is negative.
